# Supplementary material for: Hepatoprotective effects of Cassiae Semen on mice with non-alcoholic fatty liver disease based on gut microbiota
Source: Commun Biol. 2021 Dec 3;4:1357. doi: 10.1038/s42003-021-02883-8 (PMC8642482; doi:10.1038/s42003-021-02883-8)
Supplement: Supplementary file 2 — Supplemental information [file 42003_2021_2883_MOESM2_ESM.pdf]

Supplementary Fig. 1 CSE (10 g/kg), TA (10 g/kg), RG (20 mg/kg) or AO (20 mg/kg) altered microbiota diversities in NAFLD mice.

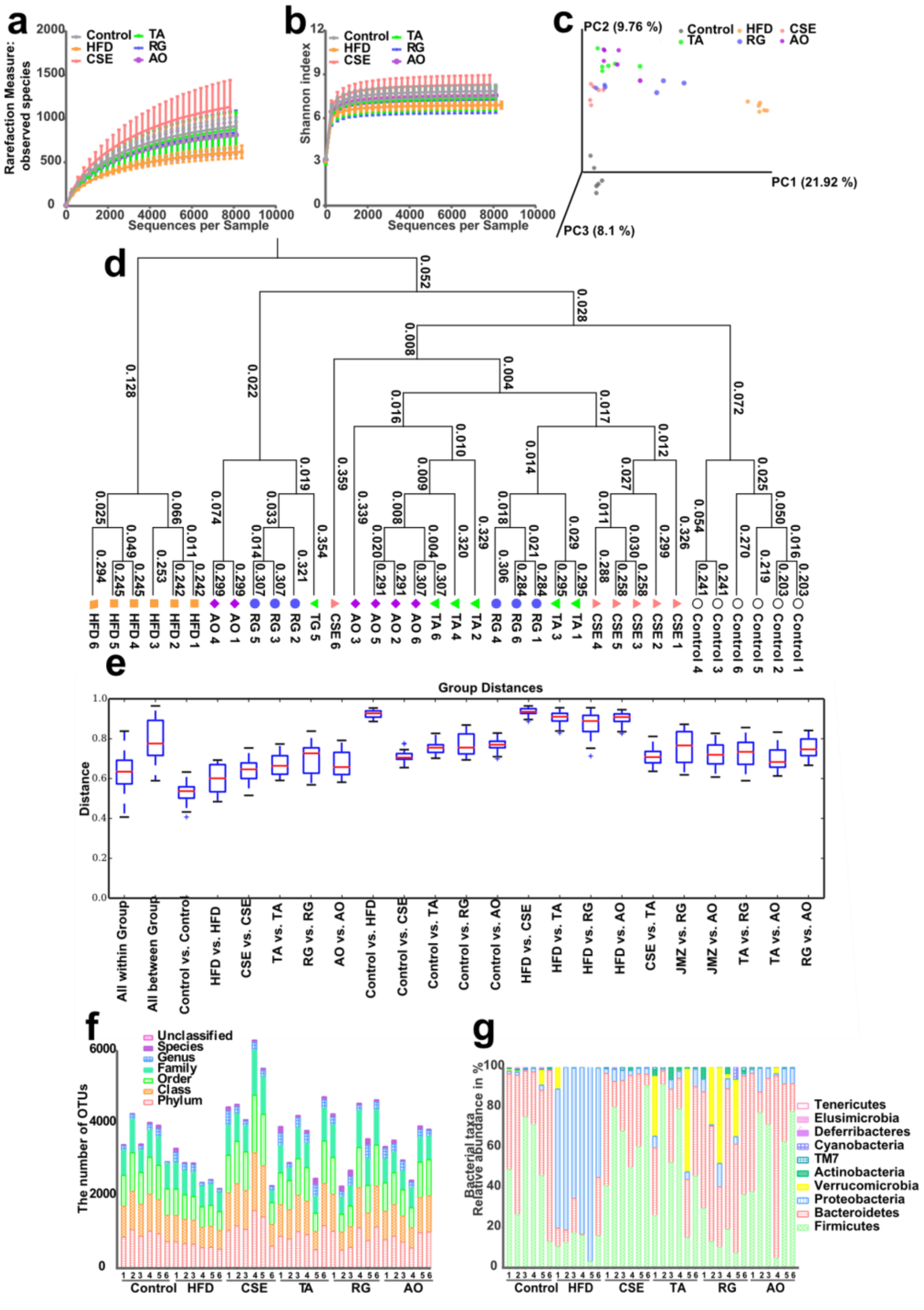

Alpha diversity analysis, for example, Rarefaction analysis (**a**) and Shannon index (**b**) of fecal samples from Control, HFD, CSE, TA, RG, and AO group mice (n=6 for each group), shown the diversity of each sample community. UniFrac-based principal coordinates analysis (PCoA) (**c**), Multivariate analysis of variance of PCoA matrix scores (**d**), and the differences of UniFrac distance between / within groups analysis (**e**), indicated the similarity of community structure among different samples. OTU classification and taxonomic identification results (**f**), taxonomic composition and abundance distribution of communities at phylum level in study groups (**g**), n = 6 mice/group.

Supplementary Fig. 2 Gut microbiota changes by FMT.

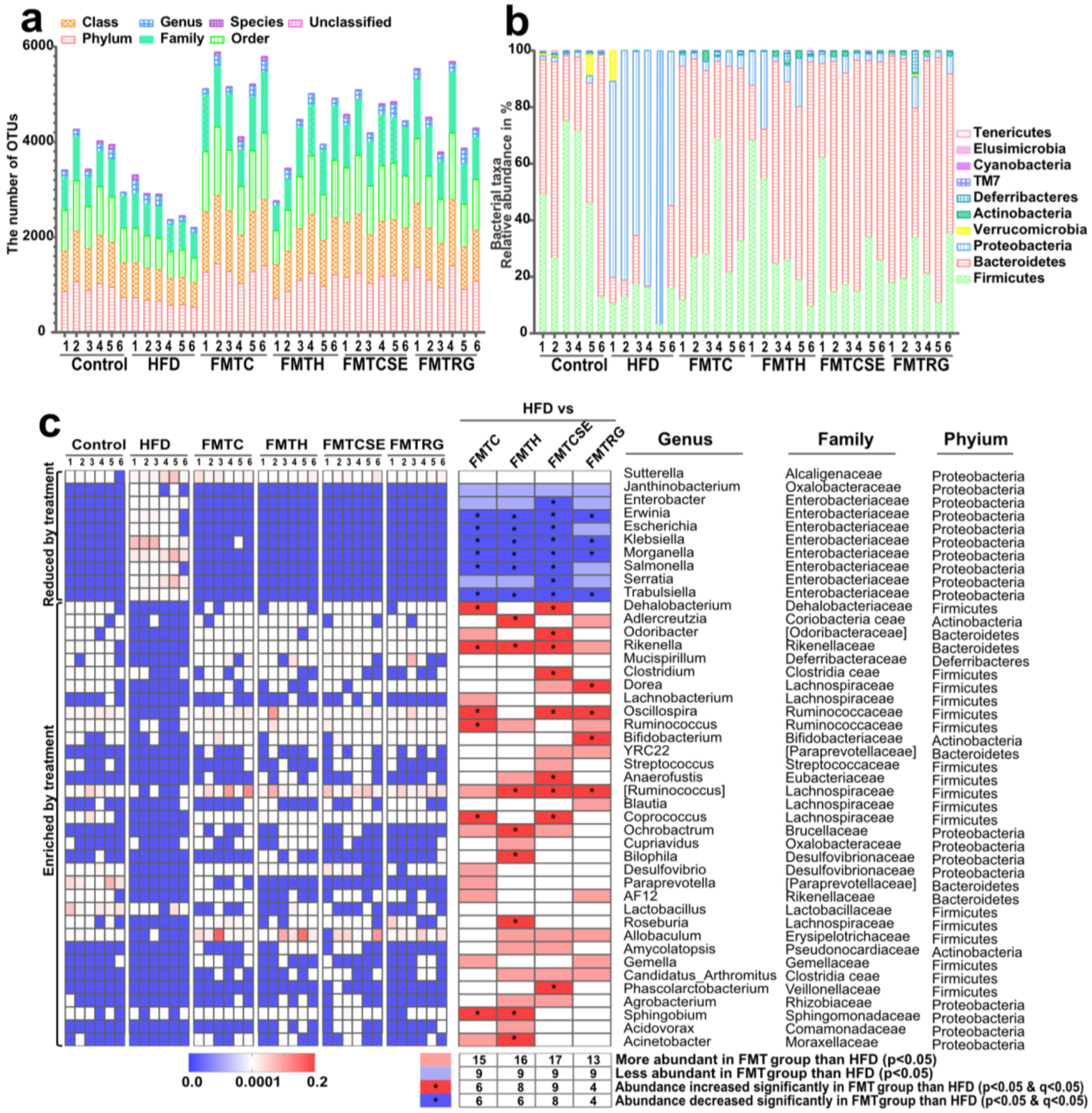

OTU classification and taxonomic identification results (**a**), taxonomic composition and abundance distribution of communities at phylum level in study groups (**b**), n = 6 mice/group. Genera changes by FMT (**c**). Heatmaps on the left show that relative abundance of bacterial genera was altered. Statistical analysis of differences between groups was performed by Metastats; in the image, red represents increased relative abundance, blue represents decreased, and * represents a significant change in genus (both P < 0.05 and Q < 0.05). The corresponding genus information is on the right.

Supplementary Fig. 3 Gut microbiota changes by antibiotic treatments.

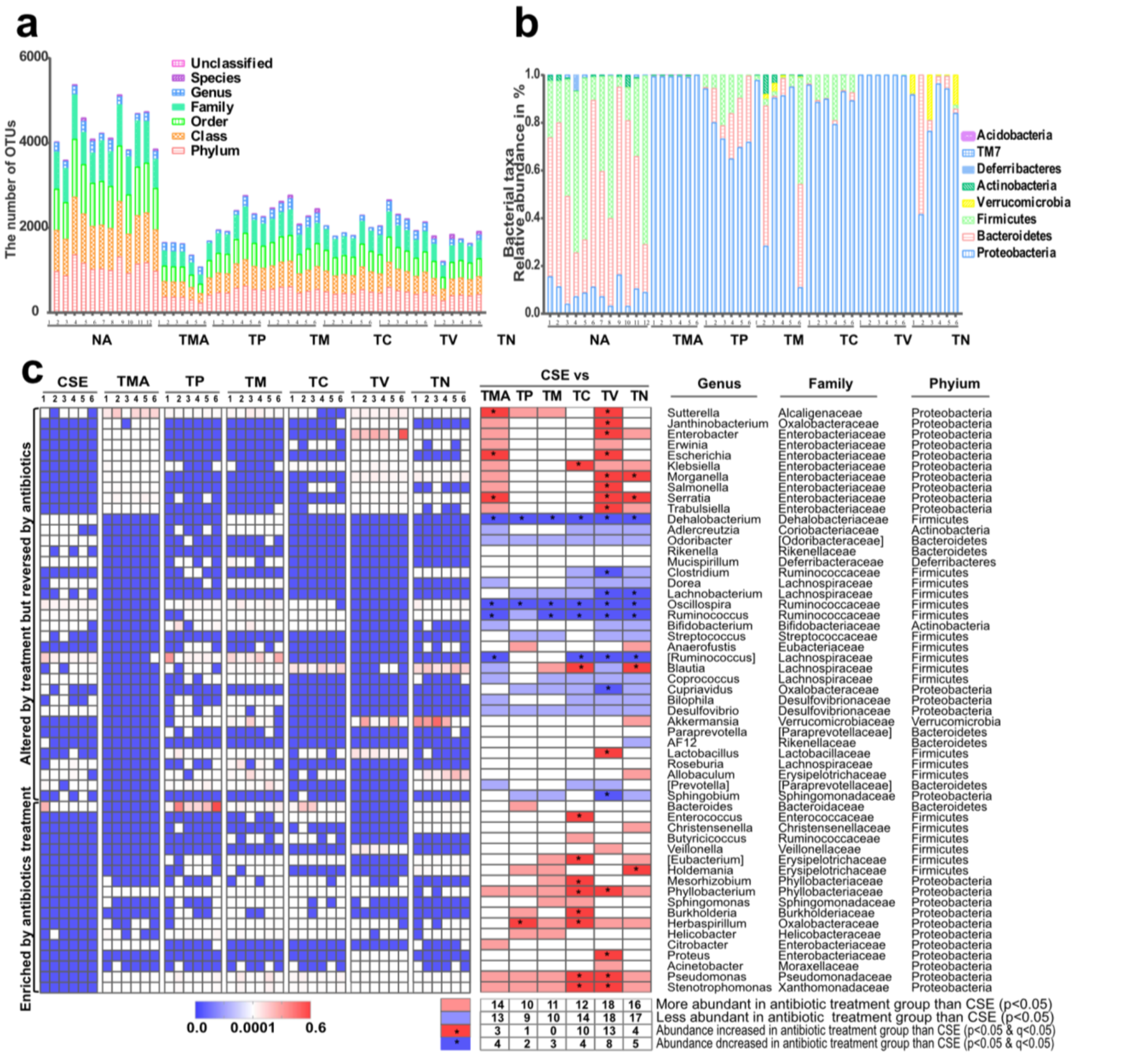

OTU classification and taxonomic status identification data of antibiotic treatment group before CSE administration (**a**). Taxonomic composition and abundance distribution of communities at the phylum level for each antibiotic treatment group before CSE administration n = 6 mice/group (**b**). Genera changes by antibiotic treatment (**c**). Heatmaps on the left show that relative abundance of bacterial genera was altered. Statistical analysis of differences between groups was performed by Metastats; in the image, red represents increased relative abundance, blue represents decreased, and * represents a significant change in genus (both P < 0.05 and Q < 0.05). The corresponding genus information is on the right.

Supplementary Fig. 4 Effects of CSE on Glycerolipid metabolism pathway.

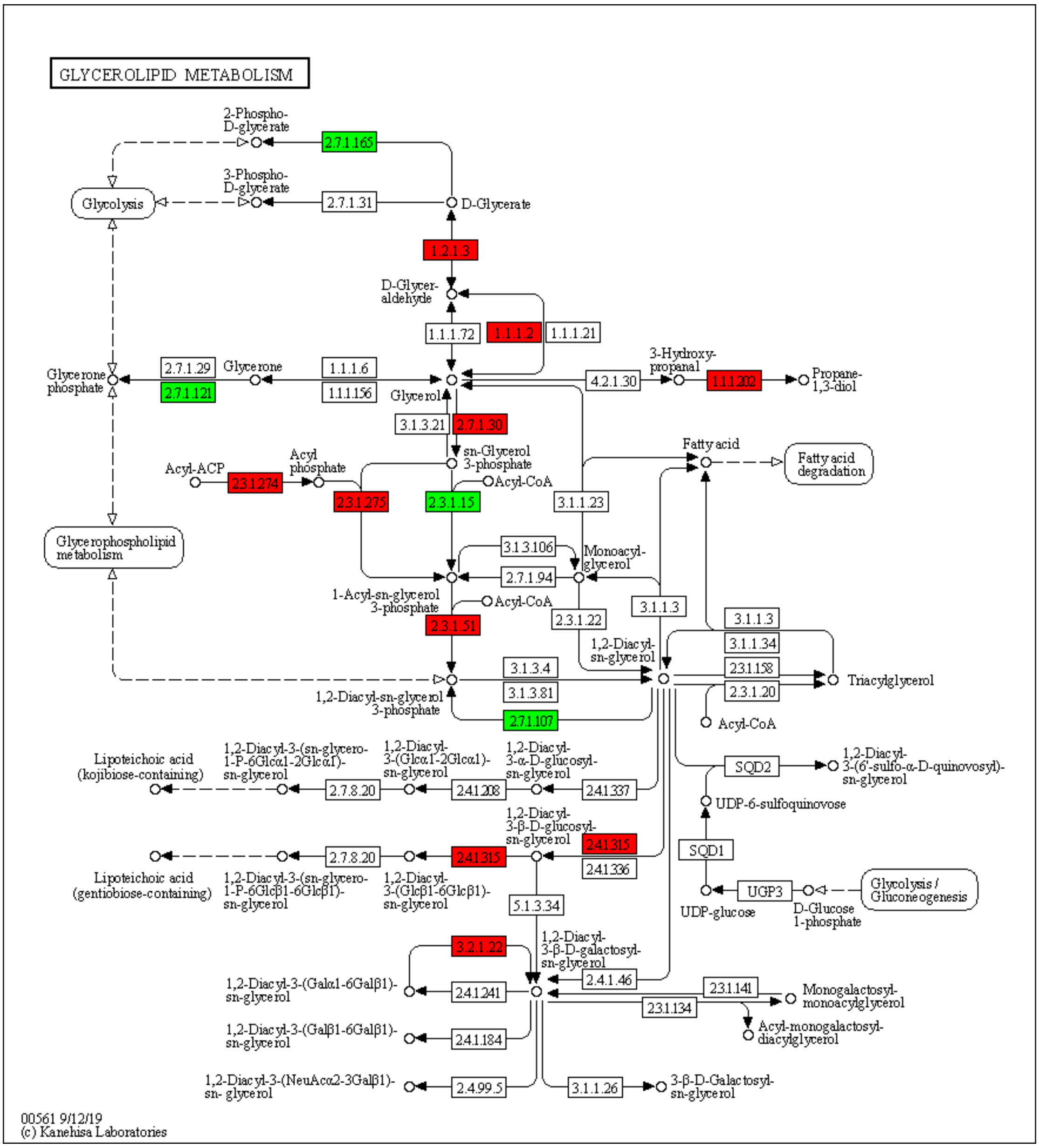

The genes marked in red means up-regulated by CSE treatment, green means down-regulated by CSE treatment, compare to HFD group.

Supplementary Fig. 5 Effects of HFD on Lipopolysaccharide biosynthesis pathway.

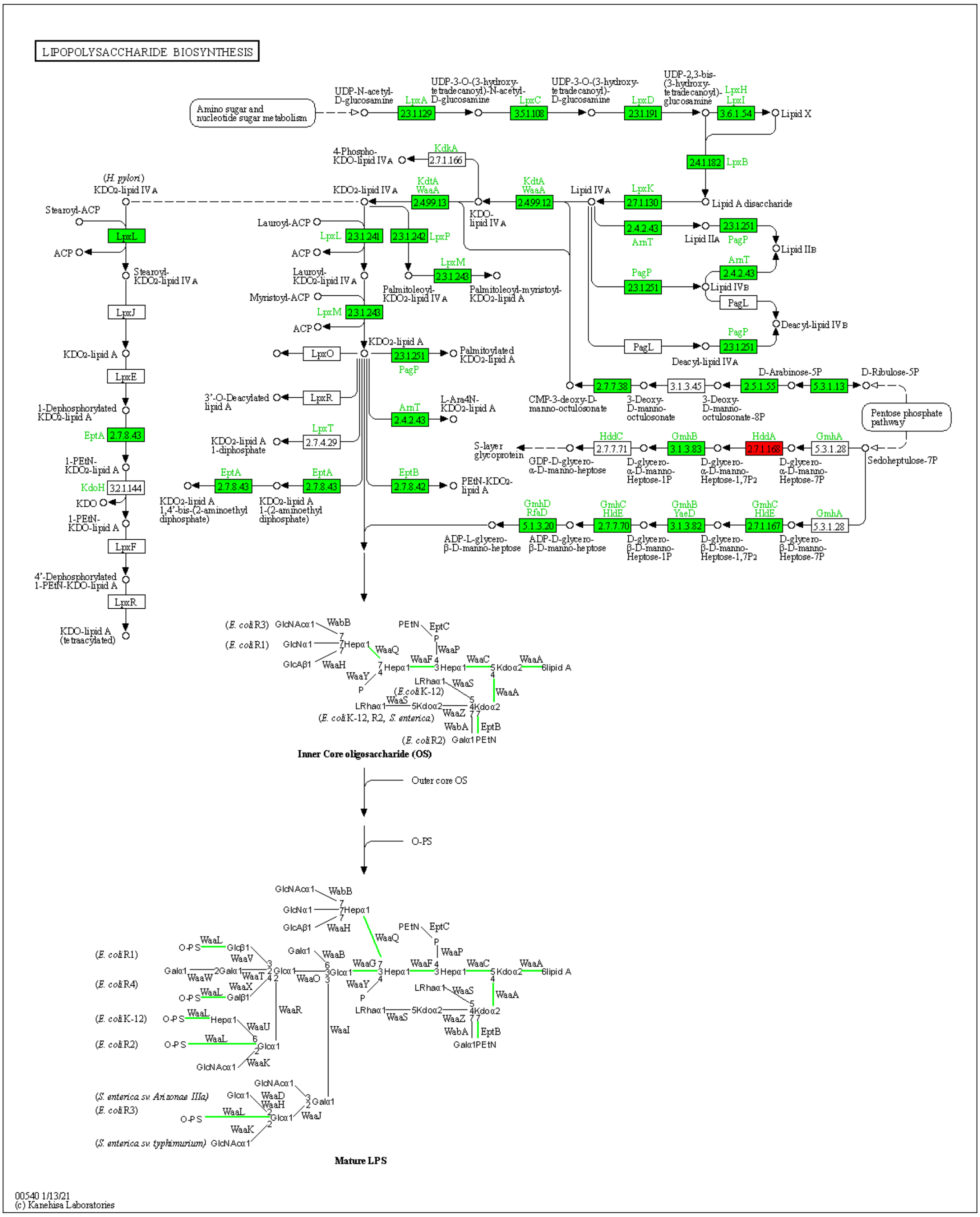

The genes marked in red means up-regulated by CSE treatment, green means down-regulated by CSE treatment, compare to HFD group.

Supplementary table 1 The forward and reverse sequences in PCR

Symbol	primer forward	primer reverse
β-Actin	GCCCTGAGGCTCTCTTCCA	GCGGATGTCGACGTCACA
Occludin	ATAATGGGAGTGAACCCGAC	CTCCTGGGGATCAACCAC
ZO-1	AACGCTCTCATAAGCTTCGTA	CTTCCCCTCAGAGACCCACA
