# Supplementary material for: Hepatoprotective effects of Cassiae Semen on mice with non-alcoholic fatty liver disease based on gut microbiota
Source: Commun Biol. 2021 Dec 3;4:1357. doi: 10.1038/s42003-021-02883-8 (PMC8642482; doi:10.1038/s42003-021-02883-8)
Supplement: Supplementary file 3 — Description of Additional Supplementary Files [file 42003_2021_2883_MOESM3_ESM.pdf]

Description of Additional Supplementary Files

File name: Supplementary Data 1.
Description: The altered KOs by HFD or extracts/compounds administration.

File name: Supplementary Data 2.
Description: The altered functional metabolic pathways by HFD or extracts/compounds administration.

File name: Supplementary Data 3.
Description: CSE, TA, RG and AO ameliorated liver weight indices and fat accumulation, liver injury and inflammation, intestinal mucosal barrier injury and altered microbiota diversity in HFD-fed mice (Raw data).

File name: Supplementary Data 4.
Description: CS effects on fat accumulation, liver injury and inflammation, and intestinal mucosal barrier injury are transferred via FMT (Raw data).

File name: Supplementary Data 5.
Description: Extracts or compounds administration ameliorate antibiotic-induced dysbiosis in HFD-fed mice (Raw data).

File name: Supplementary Data 6.
Description: Genera changes by HFD and extracts or compounds administration (Raw data).

File name: Supplementary Data 7.
Description: KEGG pathway analysis (Raw data).
